# Chemical and Wetting Analysis of the Ni-Ti Coating on SiC Improved by a 2-Step Coating-Sintering Process

**DOI:** 10.3390/ma13225235

**Published:** 2020-11-19

**Authors:** Yang Liu, Ying-Xin Wang, Qiang Yang, Fu Wang

**Affiliations:** State Key Laboratory for Manufacturing System Engineering, School of Mechanical Engineering, Xi’an Jiaotong University, Xi’an 710049, China; leoyounglab@163.com (Y.L.); 13571937318@163.com (Y.-X.W.); qiangyang@xjtu.edu.cn (Q.Y.)

**Keywords:** metal-ceramic bonding, wetting, contact angle, sintering, surface modification

## Abstract

A two-stepped coating-sintering process to prepare the qualified Ni–Ti transition applied in metal-ceramic bonding proved to be effective to improve the wetting abilities. The method was introduced in detail and compared with 2 control groups. To analyze the benefits, the morphology and composition were captured by field emission scanning electron microscopy (FE-SEM), energy dispersive spectroscopy (EDS), and X-ray diffraction (XRD). The comparisons of different coating depths and different sintering conditions were also recorded and analyzed. The influence of the EDS detecting depth was a concern in the discussions. Finally, the contact angle tests and surface energies were also estimated to verify the reliability of the transition layer. The results indicated that the coating-sintering process combined with protective sintering was preferred and necessary to increase the activeness.

## 1. Introduction

Metal-ceramic bonding involves important issues of joining, welding and surface modification. The elemental purpose to make the bonding is to develop the new structure containing both the high comprehensive properties of metals and the high wear/corrosion resistance of ceramics [1,2]. For example, the next generation turbine engine components are designed to work at high temperatures under harsh conditions, which increases the requirements of wear/corrosion resistances that metals cannot afford singly. The ceramics are usually used as the protective interface to avoid overheat and failures of the metal parts [3].

However, it is a long-standing problem to prepare qualified metal–ceramic bonding. The main reason has been regarded as the poor thermal expansion adjustments and interface combinations. A general solution is to prepare a transition layer on the ceramic–metal interface to enhance the wetting properties and reduce the interface strain [4,5,6].

In this solution, it has been reported that titanium and its alloys were often used to provide a transition layer, with high chemical activity [7,8]. Based on the situation mentioned, the nickel-titanium(Ni–Ti) transition has increasingly become a better choice, because a nickel-based composition is much more suitable to be the connection to the (nickel-based) superalloys, extending the applications of the bonding [9,10]. Some works have successfully analyzed the properties of the Ni-Ti transition layer obtained by diffusion bonding [11,12]. However, the application of diffusion bonding is dependent on the chemical compositions of the two interfaces. That is to say, using diffusion bonding to generate the Ni–Ti transition is not suitable for all metals/ceramics.

Therefore, growing Ni–Ti layer by a coating method seems to be preferred in general conditions [13]. To make the coatings denser and stronger, heat treatment should be required to promote the diffusion and bonding [14,15]. In this situation, the challenge is to avoid oxidizing at high temperatures, because the existence of oxidation was tested as a negative factor for the bonding and wetting [3,6]; the high bond energy of oxidation could sharply decrease the free energy of the residual dangling bonds on the interface. It was found that the activeness of the titanium surface can be fully damaged by an oxygen layer of only 3–5 nm thickness [16,17].

A breakthrough point is to generate the layer in a coating-sintering process. The coating step could be done at low temperature to reduce the reactions of pollution, i.e., cold spraying and magnetron sputtering. The next sintering step could be applied in a vacuum to enhance the bonding. In this process, the core heating has been designed to occur when the material has a reduced specific surface area (as a layer) than that in the droplet state (in hot spraying). It might be helpful because the pollution (including oxidation) quantity was proved to be linearly dependent on the specific surface area [18].

Consequently, to develop a universal method that is possible to benefit the application of metal-ceramic bonding, using the Ni–Ti transition layer obtained by a coating-sintering process will be of great significance. The design might decrease the risk of pollution and extend the range of choices of materials. However, there are few works concerning that situation.

To verify the design, two different coating-sintering processes were managed to generate the Ni–Ti films on the silicon carbide (SiC) substrates in this research. The surface properties of the layers were evaluated and the behaviors of the materials were also carefully characterized.

## 2. Materials and Methods

### 2.1. Preparation of the Metal–Ceramic Bonding Samples

The first coating step was done by magnetron sputtering at room temperature (25 °C). The selected ceramic substrate was a pure SiC cube of size 10 × 10 × 7 (length × width × height) mm. All the substrates were sprayed in the Z direction (selecting one surface of 10 × 10), using DICOVERY635 (DENTON, Moorestown, NJ, USA). A nominal Ni–Ti (Ti 40 at.%, Ni 60 at.%) alloy was chosen as the sputtering material. The coating depth was controlled by the equipment program, 500 nm for the following main experiment, and 400/600 nm for the comparisons.

The second sintering step was done using a TL1200 (Boyuntong, Nanjing, China) stove under the conditions of 1000 °C, 10–2 Pa (nominal vacuum) and holding 0.5 h. After heating, the samples were cooled with the furnace. Although it was reported that a 600 °C treatment was enough for the crystallization of Ni–Ti [19], the bonding temperature should be raised to 1000 °C [20] to meet the future joining/welding requirements, designed to enhance the connection force to ceramics.

During the sintering step, the samples were divided into 2 groups. One of the 2 groups should be heated together with >200 g sponge titanium laying around (protectively-sintered), another group would be done as designed (sintered). The protectively-sintered group was planned to sacrifice the added oxygen-absorbed material to prevent further oxidation of the coatings.

### 2.2. Chemical Composition, Morphology, and Phase Analysis of Different Samples

The detailed morphology of the samples above (including the states before sintering) were captured by the field emission scanning electron microscopy (FE-SEM) technology with a Hitachi S-4800 (Hitachi, Tokyo, Japan). The compositions of the interfaces were also simultaneously quantified by energy dispersive spectroscopy (EDS) detection at 15 kV. The EDS mapping was used to appraise the homogenization of the element distributions, to ensure that further analysis was valid.

The detected EDS elements were next used to estimate the X-ray signal depth based on ISO 14594:2014(E), using a EDS-Analyzer (Leo-iTech, 1.0 alpha, Xi’an, China). This analysis was used for a preliminary evaluation of the element diffusion behavior.

X-ray diffraction (XRD) analysis was needed for the samples (before and after sintering). The observations were recorded under the 2-theta of 20–80 degree, using the X’Pert Pro (PANalytica, Almelo, Holland). The semi-quantitative analysis was indicated by Jade (Materials Data, 9.0, Livermore, USA) with the full width at half the maximum method (FWHM).

All the analysis here was done for each sample, including the groups with other coating depths (400, 500 and 600 nm).

### 2.3. Contacting Angle Test and the Surface Free Energy Estimation

The best way to evaluate the quality of the surface modification was to check the surface activeness. In other words, an estimation of the surface free energy could be helpful. The most widely applied method was to measure the contact angle of pure water at room temperature (25 °C). The distilled water contact angle tests were used to evaluate the surface activities of the coatings, in this case, using Streampix (NorPix, 3, Montreal, Canada).

According to the known Young’s equation, the contact angle, *θ*, can be verified by Equation (1) [21].

(1) $$\gamma_{sg}=\gamma_{sl}+\gamma_{lg}\cos\;\theta$$

In Equation (1), *γ_sg_*/*γ_lg_* is regarded as the surface energy of the solid/liquid phase, and *γ_sl_* can be regarded as the residual surface energy (after bonding and interactions) of the wetted interface. It can be concluded that a good wetting recommends the higher activeness (surface energy) of the solid. If the samples were polluted, the wetting would be weaker [22,23]. However, Equation (1) was not available to estimate *γ_sg_* if *γ_lg_* was unknown. If the unknown *γ_sg_* was regarded as a constant which was independent of the contact angle and the liquid, Equation (1) could be regarded as the following Equation (2) form [24]:

(2) $$\cos\;\theta =-1+2\sqrt{\frac{\gamma_{sg}}{\gamma_{lg}}}$$

Although Equation (2) is regarded as not correct for the general conditions, it also provides accessibility to value the solid surface energy directly [24], which can efficiently reflect the effects of the following surface modification. The value of the surface energy of water is 0.0755 J/m^2^ [25].

## 3. Results and Discussions

### 3.1. Typical Morphology of the Coated Samples Before and After Sintering

**Figure 1:**
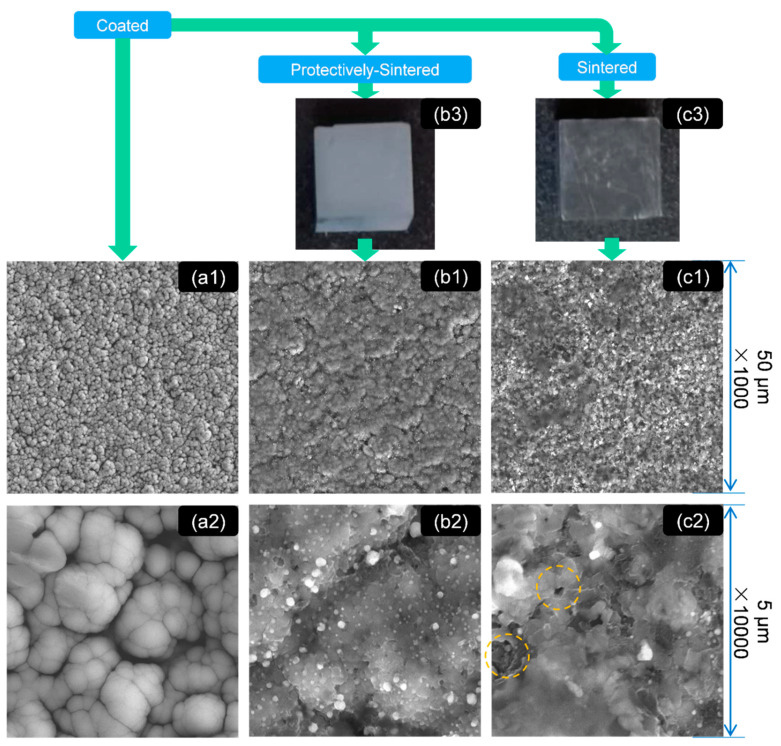
The morphology as shown by field emission scanning electron microscopy (FE-SEM) of the samples at different scales: group (**a**) the SiC substrate coated with Ni-Ti only, (**a1**) ×1000, (**a2**) ×10000; group (**b**) the protectively-sintered (sintered together with the oxygen-absorbed material) surface of (**a**), (**b1**) ×1000, (**b2**) ×10000; group (**c**) the normally-sintered surface of (**a**), (**c1**) ×1000, (**c2**) ×10000; (**b3**) the optical surface quality of the protectively-sintered substrates; and (**c3**) the optical surface quality of the sintered substrates.

**Figure 2:**
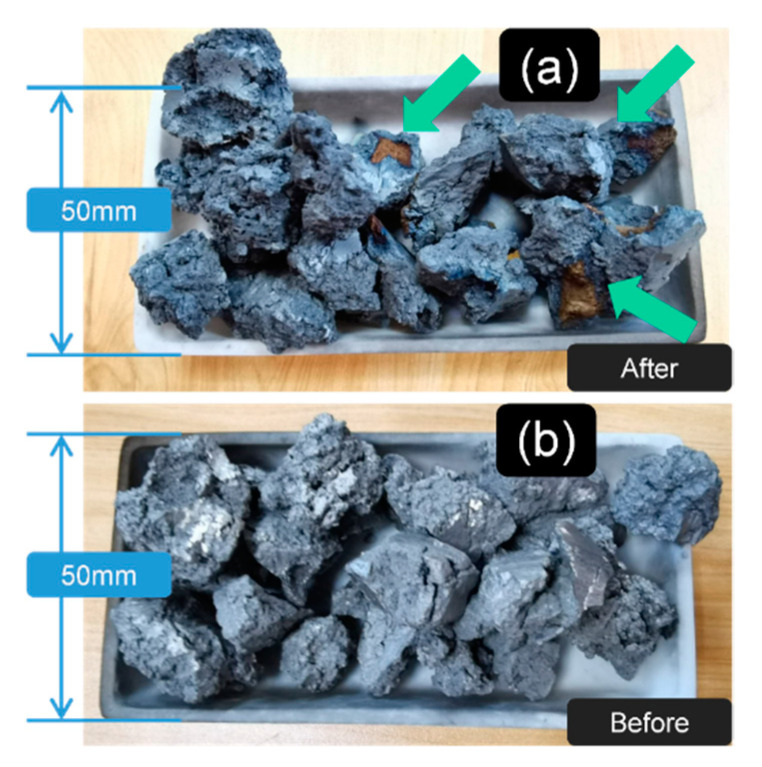
The morphology of the fed sponge titanium showed that the oxygen-absorbed metal could be polluted with the typical oxidation color even in the high vacuum environment; the result also indicated that it was necessary to add the sponge titanium in the heat-treated process: (**a**) the sintered state; and (**b**) the original state.

The surface qualities (FE-SEM scope) of the samples before and after sintering (including the protectively-sintered samples heated together with sponge titanium) can be obtained in Figure 1. The added sponge titanium before and after heating is shown in Figure 2.

In Figure 1b3,c3, it can be concluded that the surface sintered together with the oxygen-absorbed material (affording more surface to reaction [18]) was much brighter and clearer than the unprotected group. This result also matched the surface color change in Figure 2 after sintering.

Figure 1a1,a2 shows that the as-received coatings were filled with cotton-shaped structures with diameters of about 500 to 2500 nm. The cotton structure is presented with a round shape and smooth boundary/interface, which indicates that the as-coated structure must be loose. This phenomenon might prefer the explanation that the as-coated layer was formed only by agglomeration (to decrease the surface energy), so the bonding might be weak. Furthermore, the coating was sputtered at room temperature, which indicated that the diffusion would be also weak in this step.

After sintering, the characteristics of morphology were changed obviously with significant differences. The protectively-sintered samples exhibited a smoother surface than the normally-sintered group. The gullies in the as-coated sample surface also coalesced after sintering. In (×1000 scale) Figure 1a1,b1, it seems that the protectively-sintered surface partially inherited the cotton characteristics after heating, but the diameters are enlarged to 20–30 μm and the macro morphology seemed to be flatter. This result indicates that the protectively-sintered films are denser, formed by the reducing trend of the surface energy of the solid.

However, the cotton-shaped characteristic disappeared in the normally-sintered (without the protective metal) sample in Figure 1c1. The sintered surface without protection, shown in Figure 1c1, was filled with many irregular ripples and joined with holes, marked yellow in Figure 1c2. The hole structure was also found in Figure 1c1 in ×1000 scale. These results imply that when sintered without protection, the film structure would become porous, being not as dense as the protective sintering.

In Figure 1b2,c2 it is clear that many of the precipitated phases of 50–500 nm diameters were also generated after sintering. The precipitated phases created in the protectively-sintered group were distributed more homogeneously than the normally-sintered group. It might be the reason that the precipitation of the normally-sintered group was disturbed by the reactions of pollution. When polluted, the composition of the outer layer of the materials should be different, which reasonably influenced the diffusions.

In summary, the morphology of the coatings obtained by protective-sintering was much cleaner than the comparison. The purpose of adding reactive metals was effective. After sintering, the morphology of the protectively-sintered group seemed to be denser than that of the others.

### 3.2. Chemical Composition Changes Before and After Sintering

**Figure 3:**
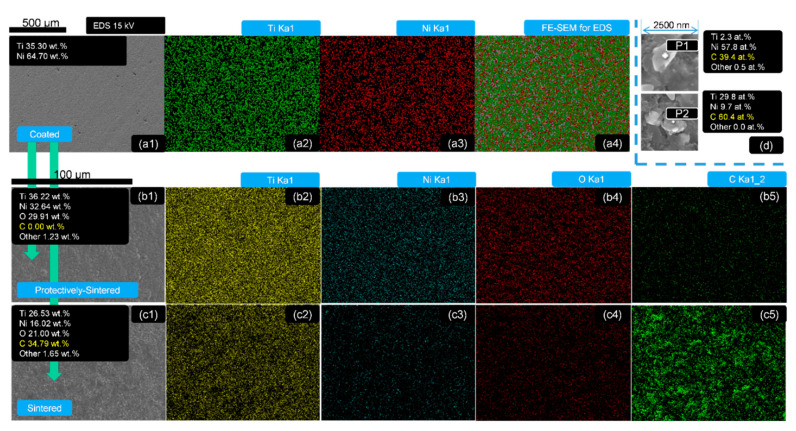
The EDS mapping (Ti, Ni, O, C) and detection of the samples of 500 nm depth: group (**a**) as-coated, (**a1**) SEM photograph, (**a2**) element Ti distribution, (**a3**) element Ni distribution, (**a4**) EDS mapping for all elements; group (**b**) protectively-sintered, (**b1**) SEM photograph, (**b2**) element Ti distribution, (**b3**) element Ni distribution, (**b4**) element O distribution, (**b5**) element C distribution; group (**c**) sintered, (**c1**) SEM photograph, (**c2**) element Ti distribution, (**c3**) element Ni distribution, (**c4**) element O distribution, (**c5**) element C distribution; (**d**) chemical compositions of the typical precipitation EDS with 2 points selected from all the sintered (protective-sintering included) samples.

To analyze the distributions of the elements of the films, EDS tests were needed. The EDS mappings of the as-coated, sintered and protectively-sintered samples of 500 nm depth are recorded in Figure 3. The typical precipitation was also evaluated by EDS in Figure 3d.

Figure 3 illustrates that all the main elements (Ni, Ti, Si, C, O) were distributed homogeneously before and after sintering (including the protective sintering). The uniform EDS mapping tests were necessary to ensure the detecting zone of the following analysis of phases and chemical compositions were reasonable.

The chemical contents were varied at different heat-treated states. The detected EDS chemical contents of the selected zones are shown in Table 1 and Figure 3 (for 500 nm samples). 

For example, the contents of the as-coated samples (500 nm) were about 64.7 wt.% Ni and 35.3 wt.% Ti (too much Ni detected); after protective sintering (500 nm), oxygen (29.91 wt.%) was added into the main elements group and the fractions of Ni and Ti seemed to be balanced at 32.54 wt.%, 36.22 wt.%, respectively. The most important result was that the detected ratio of oxygen to metal was reduced in the protective sintering. This ratio was used to directly compare the oxygen changes of the Ni–Ti coatings to avoid data interference, because a large quantity of carbon was discovered from the normally-sintered group. This result shows that carbon could be another pollution source for sintering the coatings with a small surface area; it might come from the vacuum pump (oil), which was not obvious for this common component (with a huge surface area), but it was sensitive to the small surface samples. Furthermore, the EDS compositions seemed to be independent of the coating thickness, because the results with varying coating depth were close to each other, without a significant changing trend.

The typical 2 precipitations (from all the sintered samples including the protectively-sintered group) were identified as carbide (including Ti and Ni), shown in Figure 3d. This could be regarded as the indirect evidence of the reactions of the metal-ceramic bonding because the outer surface of the layer should be a lack of carbon (for the protectively-sintered group) and the bright spots in the EDS figures in Figure 1 should be the results of a long-range diffusion.

To explain the differences in the EDS compositions before and after sintering, the influences of the detecting depth for EDS should be concerned. It should be proposed that the EDS signals (reflecting X-ray) were generated strongly related to the atomic number of the samples. The heavy elements (with higher Kα energy levels) usually showed higher absorption ability of electron beams and X-rays; thus, the detecting depth of the heavy elements would be shorter.

**Figure 4:**
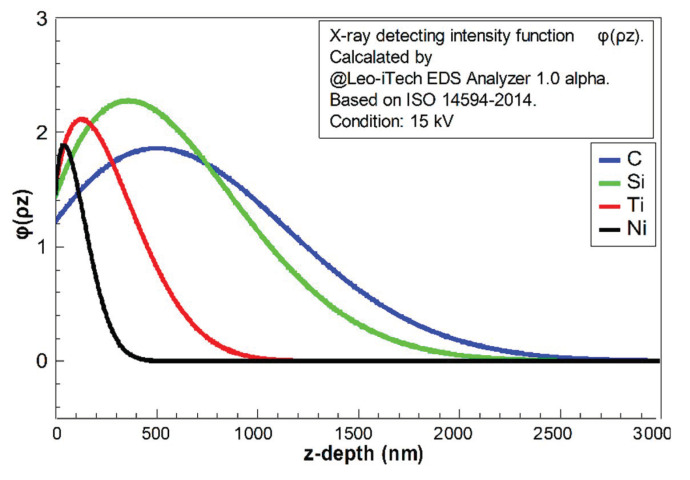
The relative intensity integral of X-ray with depth (z to infinity) calculated using the Leo-iTech EDS Analyzer 1.0 alpha for C, Si, Ti, and Ni, based on ISO 14594-2014, at voltage 15 kV.

**Figure 5:**
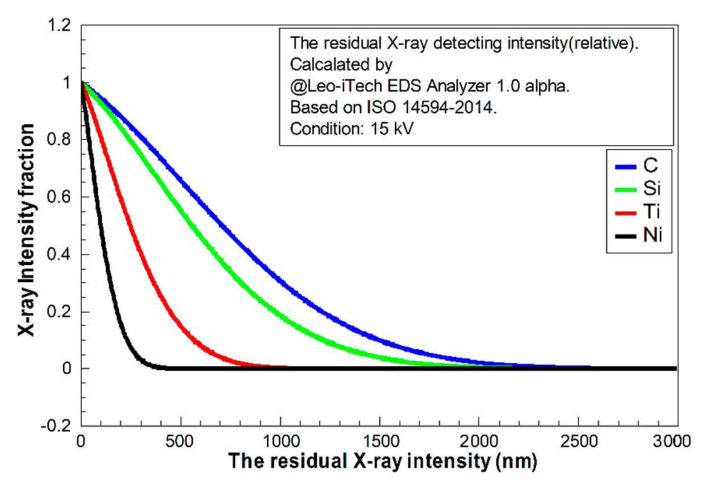
The relative intensity integral of X-ray with depth (z to infinity) calculated using the Leo-iTech EDS Analyzer 1.0 alpha for C, Si, Ti, and Ni, based on ISO 14594-2014, at voltage 15 kV.

The reflecting X-ray quantity contributed by each “layer” perpendicular to the z-direction can be estimated by the function *φ*(*ρz*). In this case, *ρ* is the density of material and *z* is the depth. The intensity of the generated X-ray is not monotonically increasing or decreasing with an increment of depth, because the X-ray generating zone was usually a drop-shaped zone under the electron beam bombardment. Therefore, the area of each generating “layer” would be increased firstly and then decreased with increment of depth, affecting the shape of function *φ*(*ρz*). The X-ray intensity should be decreasing with increasing depth by absorption [26]. According to ISO 14594-2014, *φ*(*ρz*) could be calculated. The studied pure elements in this case (C, Si, Ti, and Ni) were calculated and are shown in Figure 4 and Figure 5; these results were found using the Leo-iTech EDS Analyzer 1.0 alpha.

**Figure 6:**
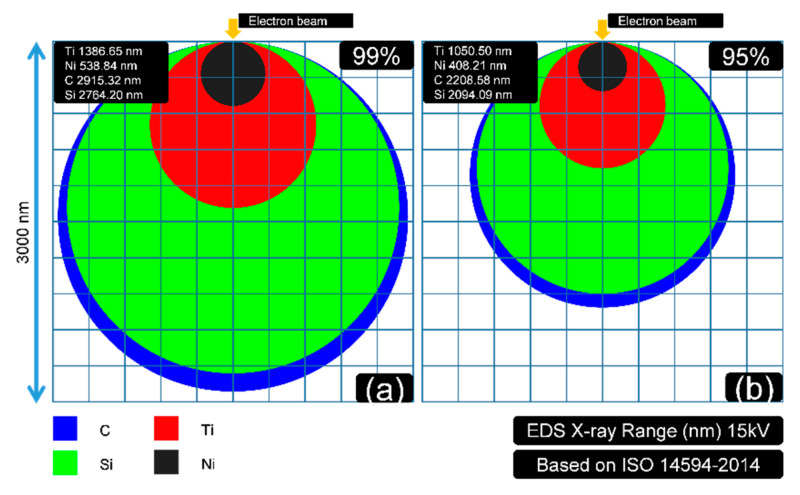
The illustration (X-ray zone was regarded approximately as a sphere) of the detected X-ray depth for each element (C, Si, Ti, and Ni): (**a**) depth of 99% of the X-ray signal detected; (**b**) depth of 95% of the X-ray signal detected.

In Figure 4, it is clear that the light elements (C and Si) had much longer “tails”. The decay of the X-ray in light-element composition was weaker. It implies that the detected quantities of light elements should be much higher than the heavy elements in EDS at the same voltage. The fractions of residual X-ray (from z to infinity) for each element are shown in Figure 5. This indicates that the detecting X-ray signal intensity had limited depth. The confirmed X-ray depth was calculated and is shown in Figure 6.

In Figure 6, the depth that 99% and 95% of the X-ray is detected are exhibited in (a) and (b), respectively. This figure was drawn with an assumption that the X-ray generating zone could be regarded approximately as a sphere. The detecting depth of EDS could be estimated as Equation (3) [27].

(3) $$Z_{m}=const.\times \left({E_{i}}^{1.7}-{E_{k}}^{1.7}\right)\times \frac{m_{a}}{\rho Z}$$

In Equation (3), *Z_m_* is the X-ray generation depth, *E_i_* is the energy of the incident electron, *E_k_* is the critical excitation energy, *m_a_* is the atomic mass of the bombarded point, *ρ* is the mass density of the bombarded point, and *Z* is the atomic number of the bombarded point.

It can be concluded that the X-ray generation depths of C and Si, in this case, were much more than Ti and Ni at 15 kV. For a 95% X-ray situation, the generation depths of the selected elements C, Si, Ti, and Ni are 2208.56, 2094.09, 1050.50, and 408.21 nm, respectively. The data (for 99% X-ray) were 2915.32, 2764.20, 1386.65, and 538.54 nm.

Combined with the previous part (EDS results), it can be concluded that the real fraction of the heavy element Ni, in this case, should be larger than the given data in Table 1, because the X-ray generating depth of Ni is much thicker than the other elements. The light elements of Si and C (including O) should not be as much as the presented data. The detected fraction of oxygen should also be enlarged (in EDS). However, oxygen could not directly fit the model in Figure 4, Figure 5 and Figure 6, because it was not reasonable to regard oxygen as solid in that situation.

The calculations in Figure 4, Figure 5 and Figure 6 could explain why the test voltage should be controlled equally as 15 kV. The changes in voltage would influence the detecting depth according to Equation (3), which contributes to the further distortions of the quantitative EDS data. The set 15 kV parameter also fitted the coating depth (400–600 nm) to the key element Ni (538.84 nm, 99%) to ensure the EDS in this part was reasonable (should have enough intensity of all the key elements).

Therefore, one could deduce the following analysis from the EDS changes before and after sintering in Table 1.

Before sintering, the detected main elements were Ni (major part) and Ti (small part), and the real percentage of Ni should be more. It indicates that Ni should be accumulated in the outer layer of the coating at first. It means that the original element distributions in the z-direction were gradient, although the distributions parallel to the layer plane were homogeneous. A possible explanation is that the sputtering efficiency of Ni was larger than Ti in this situation [28], which caused the slight segregation (in the z-direction) of sputtering. The set deposition temperature was too low to diffuse in the as-coated samples.

After protective-sintering at 1000 °C and 0.5 h, the thick films should be annealed as the diffused stable states, so the Ti/Ni ratio should be reasonable for the sputtering material.

However, the detected Ti/Ni ratio in the normally-sintered group was more than that of the protective-sintering. It might be the effect of structure (morphology). It could be found that the normally-sintered surface had been seriously polluted (oxidation), as shown in Figure 1c1, containing many porous structures (bright) at the outer interface. The sintered coatings could not be as flat as the protective group; then the porous structure might cover the signal and should have been detected from the deeper layer (the EDS results were strongly dependent on the detecting depth, mentioned above). These porous structures (outer layer) could have been more likely the active Ti than the inert Ni. It was mentioned above that the EDS results are strongly dependent on the detecting depth.

Although the EDS compositions of the samples of different states were complex, the core trend was that the metal elements should have a diffusion of homogenization in the z-direction after heat treatment, and the Ti/Ni ratio detected should be increased after sintering. The ratio of oxygen to metal was also reduced in the protected group.

### 3.3. Phase Changes

**Figure 7:**
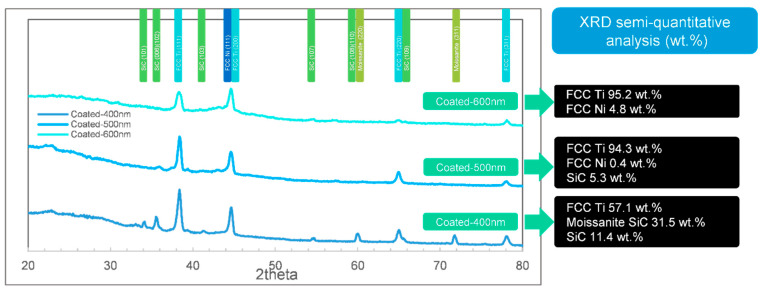
The and X-ray diffraction (XRD) phase identifications (semi-quantitative analysis) by full width at half the maximum method (FWHM) of the as-coated samples ranging from 400 to 600 nm.

**Figure 8:**
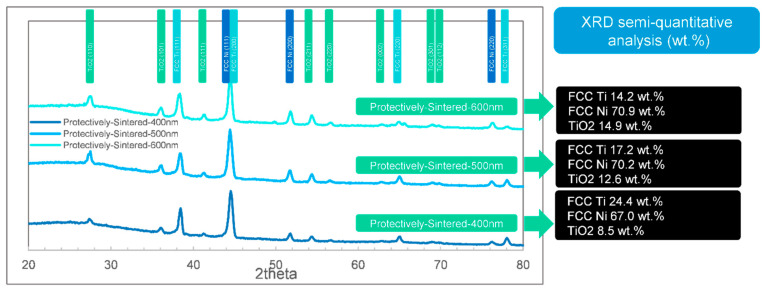
The XRD phase identifications (semi-quantitative analysis) by FWHM of the protective-sintered samples ranging from 400 to 600 nm.

**Figure 9:**
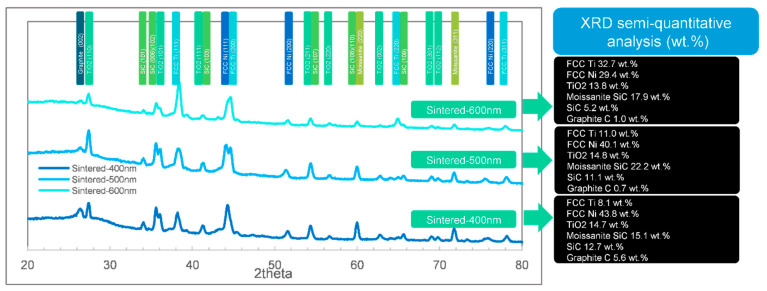
The XRD phase identifications (semi-quantitative analysis) by FWHM of the normally-sintered samples ranging from 400 to 600 nm.

To analyze the structures of the films in detail, the XRD results are essential. The XRD tests (including semi-quantitative analysis of phases) of the 3 different samples (coated, sintered, and protectively-sintered) with depths ranging from 400 to 600 nm are illustrated in Figure 7, Figure 8 and Figure 9.

General speaking, it shows that the as-coated samples lacked an Ni phase in XRD (titanium rich). The compositions seemed to be more reasonable in the sintered groups, and the unprotected samples showed much more complex phases. The influences of depth were weak for these samples.

**Figure 10:**
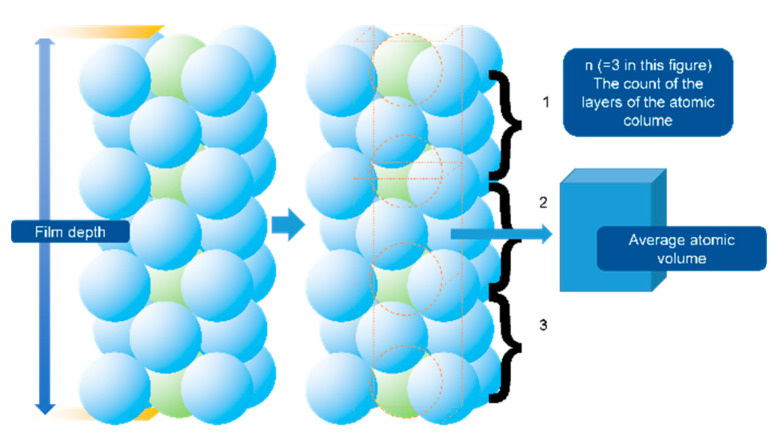
The instructions of the *ΔG* of the atomic column of the depth direction [30].

In Figure 7, the results show that the main phase of the as-coated Ni-Ti film (400–600 nm) is titanium, identified as the Fm-3m (2 2 5) space group, face-centered cubic (FCC) structure. The patterns present well-defined peaks at 2*θ* = 38.37° and 2*θ* = 44.6°, which correspond to the typical (1 1 1) and (2 0 0) planes of the face-centered cubic (FCC) structure [29]. The FCC structure is rare for titanium, but it was reported that the existing was reasonable in the thick film condition [30]. The FCC phase could exist in film <720 nm and could be stable if <144 nm. The reported specific surface energies of the FCC and hexagonal closed-packed (HCP) structures were 1.99 and 2.47 J/m^2^, respectively. The deduced strain energies of the FCC and HCP structures were 1.014 × 10^6^ and 21.618 × 10^6^ J/m^3^, respectively. The total free energy change (*ΔG*) of the z-direction atomic column Ti film from FCC to HCP can be described as Equation (4) [30]. The schematic diagram of this differential element is shown in Figure 10.

(4) $$\Delta G=-n\times 1.12\times 10^{-20}+n\Omega \left(S_{hcp}-S_{fcc}\right)+\left(\frac{n\Omega }{t}\right)*\left(\gamma_{hcp}-\gamma_{fcc}\right)$$

In Equation (4), *t* is the depth of the film, *n* is the total crystal plane (being parallel to the film surface) number in the selected atomic column, and *Ω* is the average atomic volume (regarded similar for the HCP and FCC states). The constant 1.12 × 10^−20^ is the recommended difference in bulk free energy (FCC-HCP) J/atom.

Hence, the strain energy of the FCC structure should be lower, but the specific surface energy was higher than the general HCP structure. The forming of the stable FCC phase required that *ΔG* should be positive, which indicated that it was possible to keep the stable FCC phases if the film depth was under a critical diameter. Therefore, the thin film structure requiring the higher surface area and lower volume should be the FCC structure to reduce the free energy.

However, the nickel phases of the as-coated samples were not obvious in Figure 7. The nickel phase seemed to be not crystallized [19]; the reason might be that the sputtering temperature was set too low.

It was also found that the SiC phases (including moissanite SiC) could be detected in 400–500 nm groups of the as-coated samples. 42.9 wt.% SiC (including moissanite SiC) was found in the 400 nm group and 5.3 wt.% in the 500 nm group, by FWHM. However, the 600 nm group detected few SiC. It showed that the detected SiC sharply decreased while the coating depth increased, nonlinearly. Therefore, the possible explanation is that the as-coated films < 500 nm depth were not compact, just as shown in Figure 1a2. This result indicates that the coating of Ni–Ti should be deposited at least 600 nm to be qualified.

After heat-treated, all the samples showed a majority of Ni phase of FCC characteristic and Ti in FCC structure and oxide, which matched the EDS results in Table 1. The available phases were FCC Ti, FCC Ni, and TiO_2_, other existing phases (graphite, SiC, and moissanite SiC) should be interferences from sintering or substrates.

After protective sintering in Figure 8, the analyzed pure Ni was 67.0 to 70.9 wt.%, and the pure Ti was 14.2 to 24.4 wt.% (except the part of Ti in TiO_2_). The data are reasonable compared to the EDS results in Table 1 because the Ti/Ni ratio in Table 1 should be lower than the real values, according to the X-ray generating differences mentioned in Section 3.2. Thus, the contents of XRD seemed to be more reasonable than EDS in this case. It was also presented that the fraction of TiO_2_ increased with the coating depth increment. Thus, to obtain a well-active transition layer, a thinner coating seemed to be more efficient. Furthermore, it was fortunate that all the protectively-sintered samples showed few SiC signals because the coatings had been mostly compact, which matched the morphology in Figure 1b1.

Thus, the most balanced coating depth in this situation could be concluded to be 500 nm: 400 nm could bring the risk of being uncompacted while 600 nm would cause more oxidation.

For the normally-sintered sample in Figure 9, the phase composition was complex. The SiC phases (including moissanite SiC) were found in all the coatings ranging from 400 to 600 nm. Compared with the XRD result in Figure 7 (SiC only appeared in the 400 and 500 nm groups) and typical morphology in Figure 1c1, one might conclude that the unprotected samples were oxidized with the mentioned porous structures (in Section 3.1 and Section 3.2), which could not prevent the X-ray touching the substrates (SiC). Since this term existed, the trend of the phase changes was not clear in this situation. Nevertheless, the XRD results were also useful to estimate the degree of pollution. The carbon fractions in Figure 9, by XRD, were lower and more reasonable than the EDS result in Table 1 because the detected EDS fraction of carbon was excessive according to Figure 4, Figure 5 and Figure 6.

In general, the sintering method of the two-step process would significantly influence the surface quality. Although a high vacuum was enough for common heat treatment, sintering for bonding should be strictly qualified. Therefore, protective sintering was recommended. The optimal coating depth was 500 nm. It was also essential that the XRD results were more faithful for the thin-film samples, especially for the light elements.

### 3.4. Wetting Behaviors and Surface Free Energy

**Figure 11:**
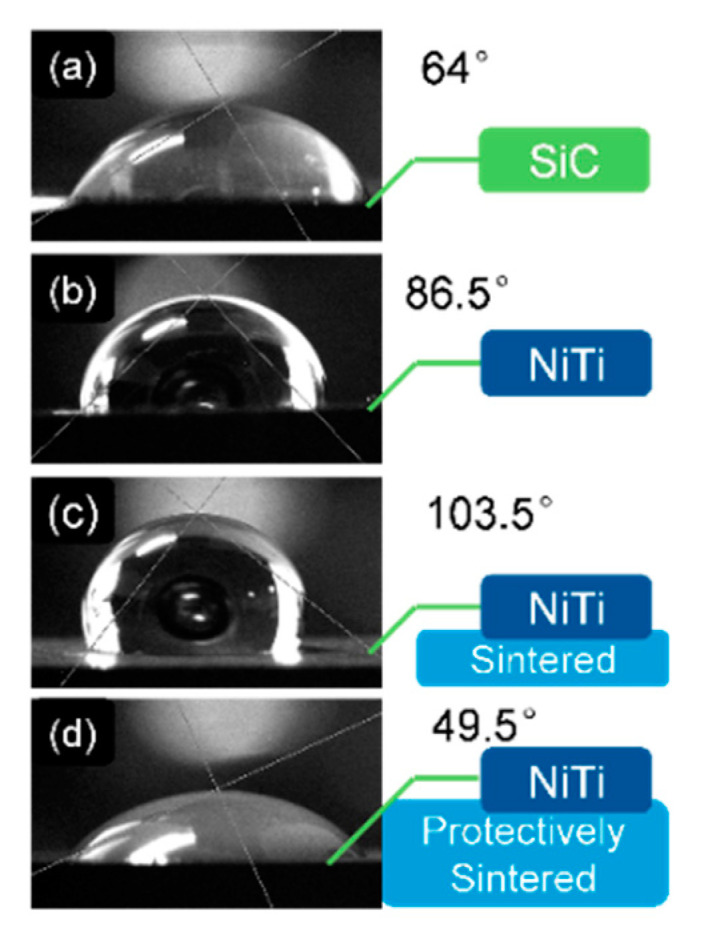
The contact angle test of the different samples with distilled water in the air: (**a**) the as-received SiC substrate; (**b**) the SiC surface coated NiTi without sintering; (**c**) the SiC surface coated NiTi after sintering (without sponge titanium); and (**d**) the SiC surface coated NiTi after the protective-sintering (with sponge titanium).

Besides the chemical analysis and the phase analysis, the contact angle test should be also included to evaluate the reactiveness of the improved interface. The wetting experiments of different surface states are shown in Figure 11.

Figure 11 shows that the contact angle between the distilled water and the naked SiC substrate is 64°. When coated with Ni–Ti, the contact angle increased to 86.5°. This increment meant that the tested Ni–Ti surface energy was reduced. Furthermore, the situation became worse after sintering without protective improvements.

Fortunately, when the sponge titanium was added in sintering, the contact angle fell from 103.5° to 49.5°. The explanation is that the dirty atmosphere (including oxygen and carbon) was absorbed by the added sponge titanium, thus the protectively-sintered Ni–Ti film was cleaner with less pollution of oxygen or carbide. The clean film contained more high-power bonds of metals, which increased the interactions of the solid/liquid surface [8,9].

The estimated surface free energies of the samples were also recorded in Table 2, according to Equation (2).

It clearly shows that only the protective sintering could achieve the purpose that the presented surface energy should be enlarged, compared with the substrates (SiC). It shows that the surface energy of the as-coated sample was lower than that of the substrate, the possible reason is that the coatings did not complete crystallization, as deduced in Section 3.3. The amorphous structure usually had lower surface energy because its surface bonding was not syntropic like crystal, with decreasing density of dangling bonds. The normally-sintered situation was worse because of the pollution.

Hence, the introduced protective-sintering was successful and necessary to prepare the metal-ceramic bonding interfaces to retain the high activeness with a better wetting behavior.

## 4. Conclusions

In this work, the preparation of the Ni–Ti transition layer by a two-stepped coating-sintering process was carefully studied. The material behaviors concerned with the surface quality and phase identifications were evaluated in detail. The following main conclusions can be confirmed in the current work:

To prepare a qualified transition layer using titanium or its alloys, one should pay attention that the thin titanium layer is an FCC structure in some conditions. The FCC titanium is stable under a critical depth for the films. The existence of the FCC structure did not seem to be sensitive, except when the thickness was increasing. Thus, future works that investigate the metal-ceramic bonding should consider this phenomenon, so as to better design the bonding.

To generate the qualified Ni-Ti transition film, the combination of low-temperature sputtering and further protective sintering is recommended. To add the reactive metals while sintering (the so-called protective sintering) was efficient and necessary to reduce the heating pollution. If not, the film became seriously oxidized at high temperatures, even under the normal sintering conditions of a vacuum of 10^−2^ Pa. In this case, only the recommended protective sintering could achieve the purpose to increase the surface activeness of the substrate (SiC cube). To make the qualified final film, 500 nm coating depth was preferred.

Although EDS detections were widely used in the film analysis, the errors should be carefully handled because the light-element signal was much higher than it would be in a real situation. The EDS signal (X-ray) intensity that was used was estimated in this research at 15 kV voltage to provide a comparison for the further studies. The XRD results seemed to be more credible to evaluate the contents.

## Figures and Tables

**Table 1:** The chemical contents of the coatings with different thicknesses and heat-treated states by energy dispersive spectroscopy (EDS) mapping.

Samples	Depth (nm)	Ti (wt.%)	Ni (wt.%)	Si (wt.%)	C (wt.%)	O (wt.%)	Other (wt.%)	Ratio of Oxygen to Metal
Coated	400	32.9	66	0	0	0	1.1	–
	500	35.3	64.7	0	0	0	0	–
	600	34.3	62.8	0	0	0	2.9	–
Protectively Sintered	400	33.4	33.87	0.36	2.81	27.95	1.62	0.4155
	500	36.22	32.64	0.42	0	29.91	0.81	0.4344
	600	33.01	31.88	0.32	4.98	29.8	0	0.4592
Sintered	400	24.65	14.82	0.38	39.59	20.57	0	0.5212
	500	26.53	16.02	0.22	34.79	21	1.43	0.4935
	600	24.84	11.72	2.22	39.92	21.3	0	0.5826

**Table 2:** The contact angles and the calculated surface free energy of the samples before and after sintering.

-	Contact Angle (°)	Surface Energy (J/m^2^)
SiC	64	0.039050706
Coated NiTi	86.5	0.021249928
Sintered	103.5	0.011091063
Protectively-Sintered	49.5	0.051352814
